# Sodium glucose cotransporter 1 ligand BLF501 as a novel tool for management of gastrointestinal mucositis

**DOI:** 10.1186/1476-4598-13-23

**Published:** 2014-02-05

**Authors:** Diego Cardani, Claudia Sardi, Barbara La Ferla, Giuseppe D’Orazio, Michele Sommariva, Fabrizio Marcucci, Daniela Olivero, Elda Tagliabue, Hermann Koepsell, Francesco Nicotra, Andrea Balsari, Cristiano Rumio

**Affiliations:** 1 Department of Pharmacology and Biomolecular Sciences, Università degli Studi di Milano, Via Trentacoste 2, 20133 Milan, Italy; 2 Humanitas Clinical and Research Center, Via Manzoni 56, 20089 Rozzano, Milan, Italy; 3 Department of Biotechnology and Bioscience & CINMPIS, Università degli Studi di Milano-Bicocca, Piazza della Scienza 2, 20126 Milan, Italy; 4 Dipartimento di Scienze Biomediche per la Salute, Università degli Studi di Milano, Via Mangiagalli 31, 20133 Milan, Italy; 5 Istituto Nazionale per lo Studio e la Cura dei Tumori, Via Venezian, 1, 20133 Milano, Italy; 6 Regina Elena National Cancer Institute, Via E. Chianesi 53, 00144 Rome, Italy; 7 BiEssseA, Via A. D’Aosta 7, 20129 Milan, Italy; 8 Department of Molecular Plant Physiology and Biophysics, Julius-von-Sachs-Institute, University of Würzburg, 97082 Würzburg, Germany

**Keywords:** Gastrointestinal mucositis, SGLT-1, Synthetic D-glucose analogs, Chemotherapy, Inflammation

## Abstract

**Background:**

Recent studies demonstrated that engagement of sodium glucose transporter 1 (SGLT-1) by orally administered D-glucose protects the intestinal mucosa from lipopolysaccharide (LPS)-induced injury. We tested whether SGLT-1 engagement might protect the intestinal mucosa from doxorubicin (DXR)- and 5-fluorouracil (5-FU)-induced injury in animal models mimicking acute or chronic mucositis.

**Methods:**

Mice were treated intraperitoneally with DXR, alone or in combination with 5-FU, and orally with BLF501, a glucose-derived synthetic compound with high affinity for SGLT-1. Intestinal mucosal epithelium integrity was assessed by histological analysis, cellular proliferation assays, real-time PCR gene expression assays and Western blot assays. Student’s t-test (paired two-tailed) and χ^2^ analyses were used for comparisons between groups. Differences were considered significant at p < 0.05.

**Results:**

BLF501 administration in mice treated with DXR and/or 5-FU decreased the injuries to the mucosa in terms of epithelial integrity and cellular proliferative ability. Co-treatment with BLF501 led to a normal expression and distribution of both zonula occludens-1 (ZO-1) and beta-catenin, which were underexpressed after treatment with either chemotherapeutic agent alone. BLF501 administration also restored normal expression of caspase-3 and ezrin/radixin/moesin (ERM), which were overexpressed after treatment with DXR and 5-FU. In SGLT1-/- mice, BLF501 had no detectable effects. BLF501 administration in wild-type mice with growing A431 tumors did not modify antitumor activity of DXR.

**Conclusions:**

BLF501-induced protection of the intestinal mucosa is a promising novel therapeutic approach to reducing the severity of chemotherapy-induced mucositis.

## Introduction

Oral and gastrointestinal mucositis are serious side effects of chemotherapy. Severe mucositis is especially common among patients who receive aggressive myeloablative chemotherapy and in patients who receive therapy for head and neck cancer [1-3]. Mucositis is a complex, multifactorial process which affects all layers of the gastrointestinal tract [4,5] and is characterized by apoptosis and reduced proliferation of epithelial cells in the intestinal crypts, villus atrophy and collagen breakdown. Mucositis impedes the efficacy of treatment protocols because it may require chemotherapy interruption, reduction in drug dosages or change to other antitumor drugs [6-8]. Treatment of mucositis is mainly symptomatic. In recent years, Palifermin has been successfully adopted for treatment of oral mucositis during chemotherapy of hematologic cancers [9]. However, except for “guidelines” for symptom management, no well-established therapeutic strategies to treat chemotherapy-induced intestinal mucositis are available [10-12]. Thus the development of an effective intervention against chemotherapy-related mucositis has high priority in oncological supportive care [13,14].

The sodium-glucose cotransporter 1 (SGLT-1) is a high-capacity glucose transporter expressed mainly in the apical membrane of epithelial cells lining the S3 segment of the proximal renal tubule and the intestinal epithelium [15,16]. SGLT-1 is the most important transporter of D-glucose and D-galactose from the small intestinal lumen into enterocytes and is upregulated in response to glucose in food [17]. Recent reports suggest additional roles for SGLT-1 which may not be directly linked to transport. For example, expression of SGLT-1 was shown to be necessary to preserve the integrity of plasma membranes and tight junctions in tubular renal epithelial cells after exposure to cisplatin *in vitro*[16,18]. Moreover, we have shown in a mouse model of septic shock that oral administration of high doses of D-glucose or the non-metabolized glucose analog 3-0-methyl-D-glucopyransoide protected the intestinal epithelium from lipopolysaccharide-induced inflammatory injury [19,20]. Based on these data, we speculated that SGLT-1-mediated signaling might be beneficial in maintaining intestinal mucosal integrity from chemotherapy-induced damage. To test this hypothesis, we examined the effects of BLF501 (formerly called “compound 5”), a synthetic compound which binds to SGLT-1 withdrawal [21], in a mouse model of doxorubicin (DXR)- and 5-fluorouracil (5-FU)-induced intestinal injury. Here, we show that BLF501-induced SGLT-1 activation protects against DXR- and 5-FU-induced injury by promoting proliferation of enterocytes and correct formation of tight and adherens junctions. BLF501 does not appear to interfere with drug antitumor activity.

## Results

### Oral administration of BLF501 protects against alterations of the small intestine induced by a single administration of DXR

The effect of BLF501 on alterations of the small intestine induced by DXR was evaluated in mice treated with: DXR alone (20 mg/kg i.p., n = 14); DXR plus BLF501 (25 μg/kg BLF501, n = 14); BLF501 alone (n = 14); or left untreated (n = 7). Half of the mice were sacrificed after 48 h and the other half, after 72 h (Table 1). The same evaluations was performed on SGLT-1^-/-^ mice after 72 h of treatment with DXR with or without BLF501 co-treatment (Table 1).

**Table 1:** **Summary of *in vivo *** **treatments**

***I*. BLF501 action/single DXR injection**								
Mouse strain	Group	Mice/Group	DXR	BLF501 25 μg/kg	Sacrifice 48 h	Sacrifice 72 h	BrdU injection	
BALB/C	UNTR	N = 7	-	-	-	N = 7	+	
	DXR	N = 14 (7 + 7)	+	-	N = 7	N = 7	+	
	DXR +BLF501 25 μg/kg	N = 14 (7 + 7)	+	+	N = 7	N = 7	+	
	BLF501 25 μg/kg	N = 14 (7 + 7)	-	+	N = 7	N = 7	+	
Mouse strain	Group	Mice/Group	DXR	BLF501 25 μg/kg	Sacrifice 48 h	Sacrifice 72 h	BrdU injection	
C57 SGLT-1 -/-	UNTR	N = 7	-	-	-	N = 7	+	
	DXR	N = 7	+	-	-	N = 7	+	
	DXR +BLF501 25 μg/kg	N = 7	+	+	-	N = 7	+	
***II*. BLF501 action/repeated DXR plus 5-FU injections**								
Mouse strain	Group	Mice/Group	DXR +5-FU	BLF501 0.25 μg/kg	BLF501 2.5 μg/kg	BLF501 25 μg/kg	Treatment days	Sacrifice day
BALB/C	UNTR	N = 7	-	-			-	Day 19
	DXR + 5FU	N = 7	+	-			Days 1, 8 and 15	
	DXR + 5FU +BLF501 0.25 μg/kg	N = 7	+	+				
	DXR + 5FU +BLF501 2.5 μg/kg	N = 7	+	-	+			
	DXR + 5FU +BLF501 25 μg/kg	N = 7	+	-	-	+		
	BLF501 25 μg/kg	N = 7	-	-	-	+		
***III*. BLF501/DXR interaction**								
Mouse strain	Group	Mice/Group	DXR	BLF501 25 μg/kg	Treatment days	Sacrifice day		
SKH-1	UNTR	N = 8	-	-	-	Day 26		
	DXR	N = 8	+	-	Days 7, 14 and 21 after cell injection			
	DXR +BLF501 25 μg/kg	N = 8	+	+				
	BLF501	N = 8	-	+				

**Figure 1:**
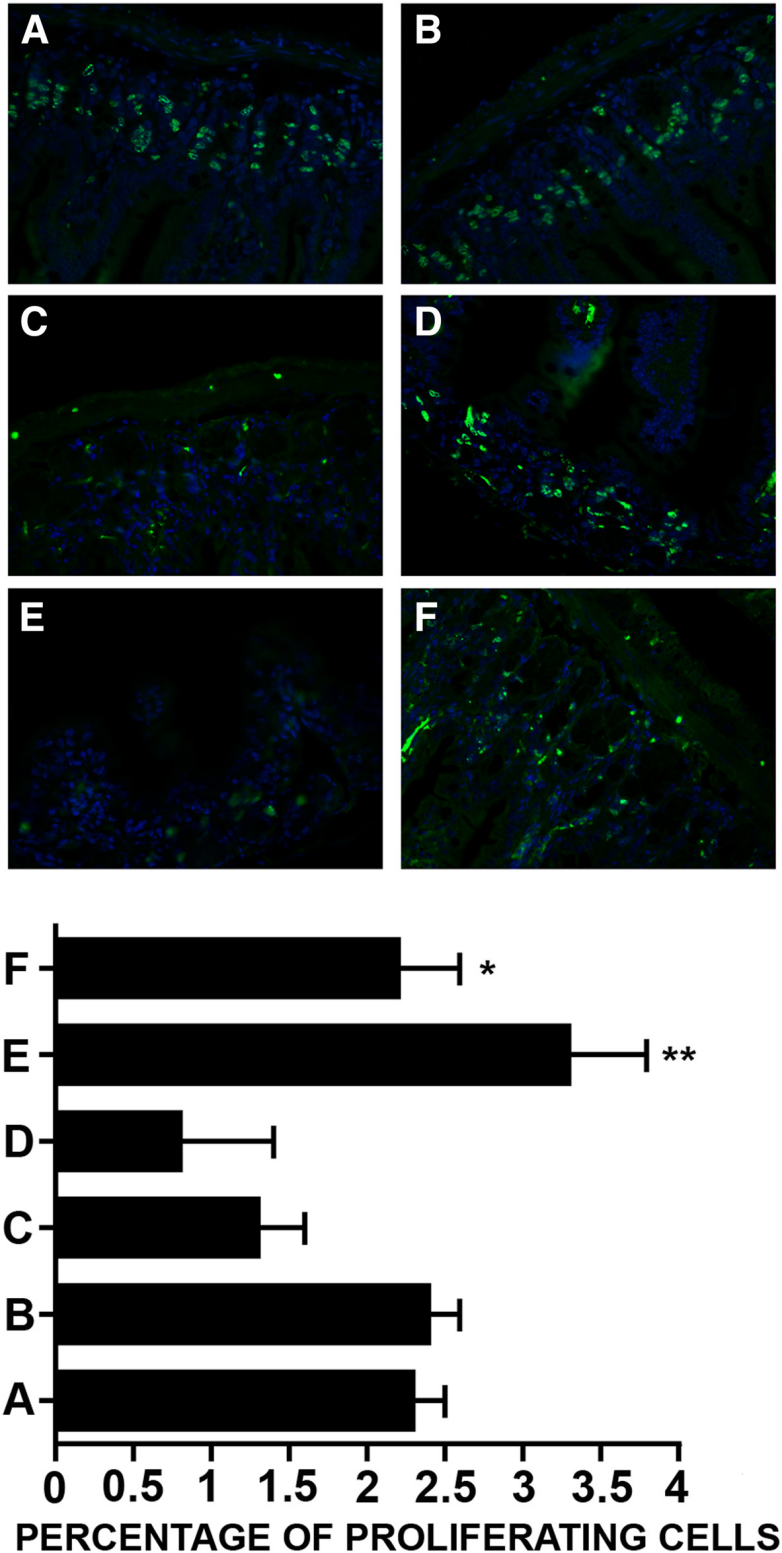
**Preservation of cell proliferation by BLF501 in small intestinal crypts of mice treated with a single administration of DXR.** Immunofluorescence assay for BrdU-positive proliferating cells was performed. DXR-induced decrease of proliferation rate apoptosis was observed at the three- to six-cell positions within crypts. Positive cells count was performed. **(A)** UNTR, untreated; **(B)** BLF501 25 μg/kg; **(C)** DXR 48 h; **(D)** DXR 72 h; **(E)** DXR + BLF501 25 μg/kg 48 h; **(F)** DXR + BLF501 25 μg/kg 72 h. DXR + BLF501 25 μg/kg 48 h *vs* DXR 48 h, ** p = 0.004; DXR + BLF501 25 μg/kg 72 h *vs* DXR 72 h, * p = 0.0282. Experiments were performed in triplicate.

Macroscopic examination of small intestine samples collected at 48 or 72 h revealed only early-stage morphological alterations, whereas immunofluorescence BrdU assay revealed a significantly reduced proliferation rate of crypt cells in DXR-treated mice [DXR 1.3 ± 0.2% at 48 h; 0.8 ± 0.6% at 72 h, UNTR 2.3 ± 2.2% at 72 h; UNTR *vs* DXR, p = 0.0086 (48 h) and p = 0.023 (72 h)] that was absent in mice treated simultaneously with DXR and BLF501 [DXR + BLF501 3.3 ± 0.5% and 2.2 ± 0.5% at 48 and 72 h, respectively; DXR + BLF501 *vs* DXR p = 0.004 (48 h) and p = 0.028 (72 h)] (Figure 1). In SGLT1-/- mice, proliferation was dramatically increased compared to that in wild-type mice, but no modification of intestinal epithelial cell proliferation was observed in samples from mice treated with BLF501 alone. BLF501 proved to be inactive in SGLT-1^-/-^ mice, with no improvements in cellular proliferation rate observed at 72 h after DXR treatment in combination with BLF501 (mean ± SD, UNTR, 11.09 ± 1.93%; DXR, 7.38 ± 2.71%; DXR + BLF501, 8.24 ± 4.59%. UNTR *vs* DXR, p = 0.0012; DXR *vs* DXR + BLF501, p = 0.3870).

**Figure 2:**
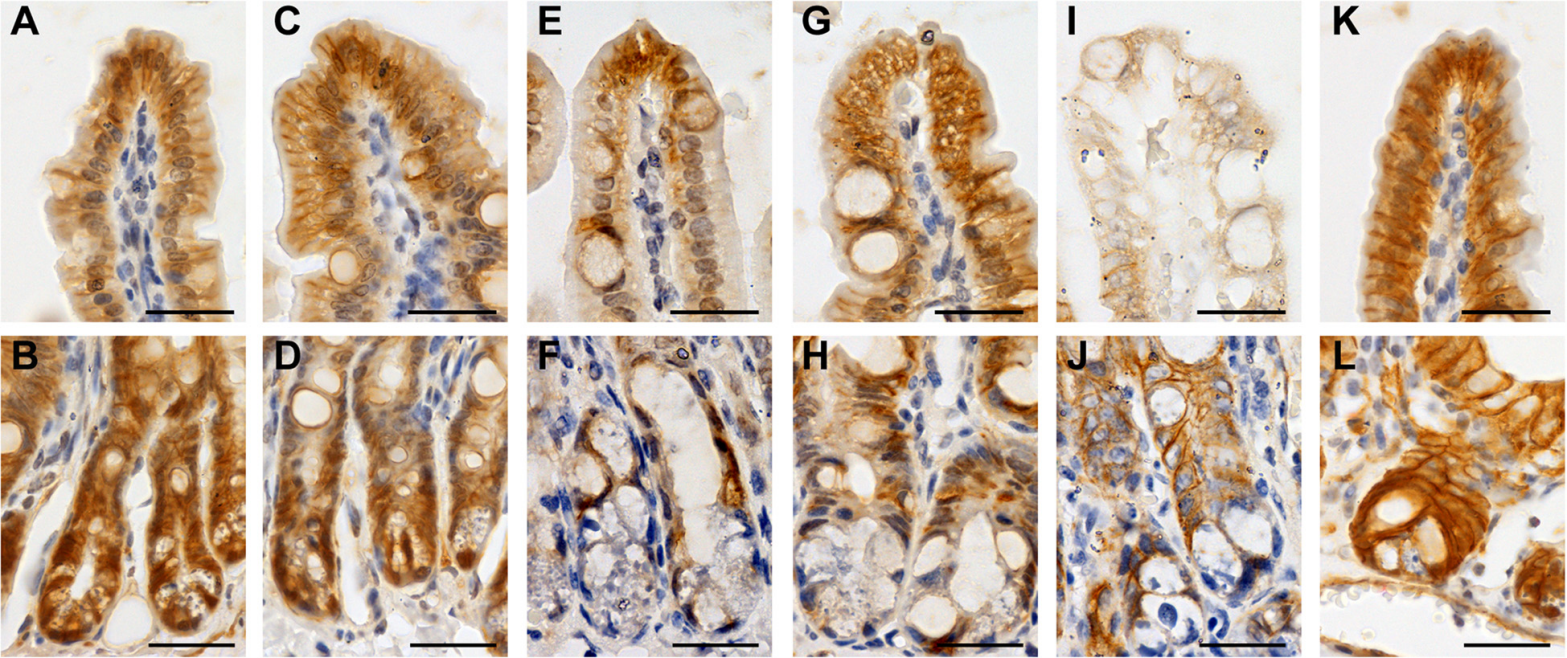
**Treatment with BLF501 maintained normal levels of beta-catenin expression in the small intestine of mice treated with a single dose of DXR.** Immunohistochemical analysis of beta-catenin: **(A, B)** UNTR, untreated; **(C, D)** BLF501 25 μg/kg; **(E, F)** DXR + 5-FU 48 h; **(G, H)** DXR + 5-FU + BLF501 25 μg/kg 48 h; **(I, J)** DXR + 5-FU 72 h; **(K, L)** DXR + 5-FU + BLF501 25 μg/kg 72 h. Bars: 20 μm. Experiments were performed in triplicate.

Expression of beta-catenin, a unique intracellular protein functioning as an integral component of the cell-cell adhesion complex and as a principal signaling protein in the canonical Wnt pathway linked to cell proliferation [22,23], was reduced at 48 h after DXR administration in the villi and, at 72 h, in both villi and crypts of wild-type mice. Treatment with BLF501 was found to maintain the physiological expression of beta-catenin (Figure 2).

**Figure 3:**
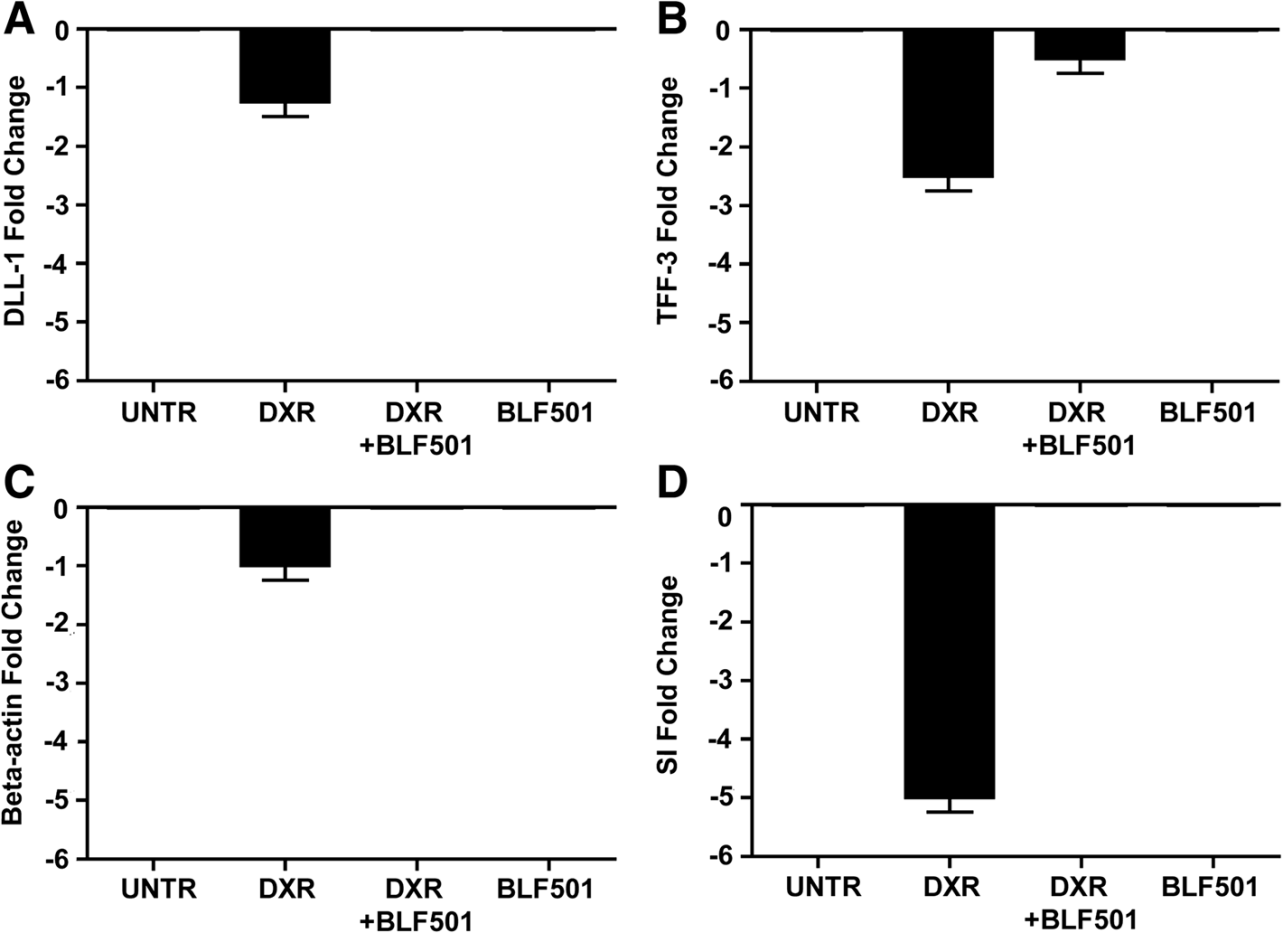
**Real-time PCR gene expression analysis of different targets implicated in the early response to tissue injury. (A)** DLL-1; **(B)** TFF-3; **(C)** beta-actin; **(D)** sucrase isomerase. Expression of all of these markers was reduced at 48 h after DXR treatment, but was similar to that in control mice after co-treatment with DXR *plus* BLF501. Error Bars means SD. Experiments were performed in triplicate.

Further analysis of the protective effect of BLF501 focused on the expression of different genes implicated in the early response to tissue injury [24], including: DLL-1, a marker of crypt cells actively proliferating in a stem cell-like manner [25]; TFF-3 and beta-actin, components of the mucus layer [26] and the cytoskeleton, respectively, and whose reduced expression mirrors decreased mucin production and alteration of cytoskeletal structure, respectively; and sucrose isomaltase, a marker for brush border integrity [24] and a key enzyme in carbohydrate metabolism, whose reduced expression mirrors epithelial damage and nutrient malabsorption [27]. Expression of all of these markers was reduced at 48 h after DXR treatment, but, after co-treatment with BLF501, was similar to that in control mice (Figure 3).

### BLF501 protects the small intestine mucosa from injury induced by repeated administration of DXR and 5-FU

**Figure 4:**
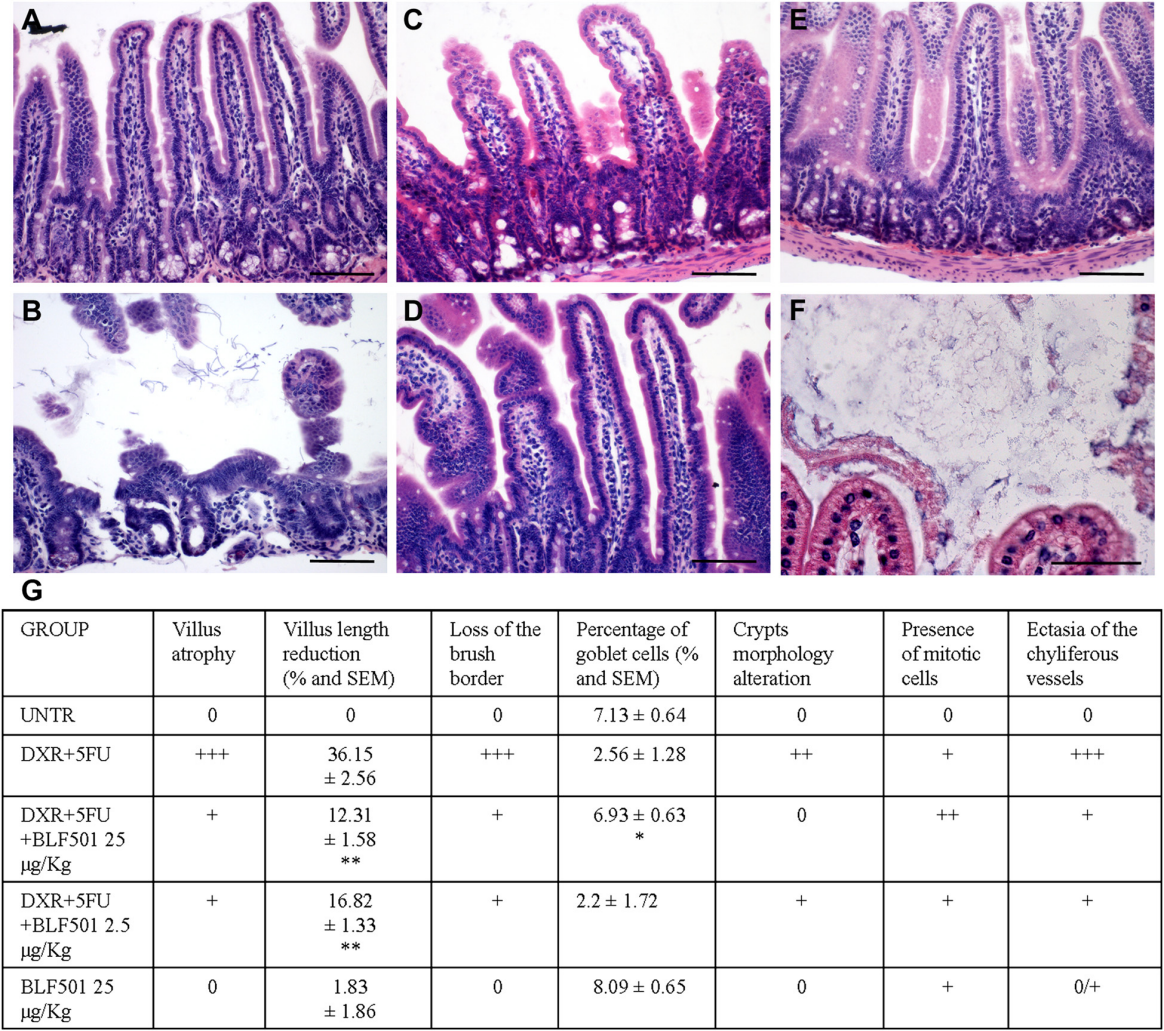
**Dose-dependent BLF501-induced protection from injury to the mucosa of the small intestine in mice repeatedly injected with DXR and 5-FU.** Histopathological detection of: **(A)** UNTR, untreated; **(B)** DXR + 5-FU; **(C)** DXR + 5-FU + BLF501 2.5 μg/kg; **(D)** DXR + 5-FU + BLF501 25 μg/kg; **(E)** BLF501 25 μg/kg; **(F)** DXR + 5-FU luminal bacterial content. **(G)** Summary of evaluated parameters. Statistical analysis: villus length, DXR + 5-FU + BLF501 25 μg/kg *vs* DXR + 5FU, ** p = 0.0014; DXR + 5FU + BLF501 2.5 μg/kg *vs* DXR + 5-FU, ** p = 0.0026. Percentage of goblet cells: DXR + 5-FU + BLF501 25 μg/kg *vs* DXR + 5-FU, * p = 0.0383. Damage score: +++ SEVERE; ++ MILD; + LIGHT; 0 ABSENT **(A-E)**. Bars: 20 μm. Results are from triplicate determinations.

We also evaluated the effect of BLF501 in a mouse model of DXR- and 5-FU-induced mucositis. The addition of 5-FU is necessary to stabilize and standardize DXR action, reducing the variability of epithelial damages in this model mimicking medium-term chemotherapy-induced effects on the intestinal mucosa, in particular, morphology and cellular population alterations and junctional systems integrity [24,28-30]. Intestinal epithelium from mice treated with the two chemotherapeutics was extensively damaged (Figure 4A-F). In particular, villi were atrophic, fused and reduced in height (-36.15 ± 2.56% *vs* untreated); epithelial cells were hyperplastic and brush borders had large areas of erosion; focal ectasia of chyliferous vessels was detectable; numbers of goblet cells were decreased (DXR + 5-FU 2.56 ± 1.28% *vs* untreated 7.13 ± 0.64%, P = 0.0065); and cells undergoing mitosis and cellular infiltrates rich in lymphocytes and plasma cells were observed. Mice treated with 25 μg/kg BLF501 showed substantial recovery from chemotherapy-induced injury to the intestinal mucosa (villus height: -12.31 ± 1.58%, p = 0.0014 *vs* untreated; goblet cells: 6.93 ± 0.63%, p = 0.0383 *vs* untreated). At 2.5 μg/kg, BLF501 improved morphological parameters (villus height: -16.82 ± 1.33%, p = 0.0026 *vs* untreated), but was ineffective against loss of goblet cells (2.2 ± 1.72%) and appearance of mitotic cells; at 0.25 μg/kg, BLF501 had no effect on any of the parameters examined. Samples from the intestinal epithelia of mice treated with BLF501 alone were identical to those of control mice. Figure 4G summarizes these results.

**Figure 5:**
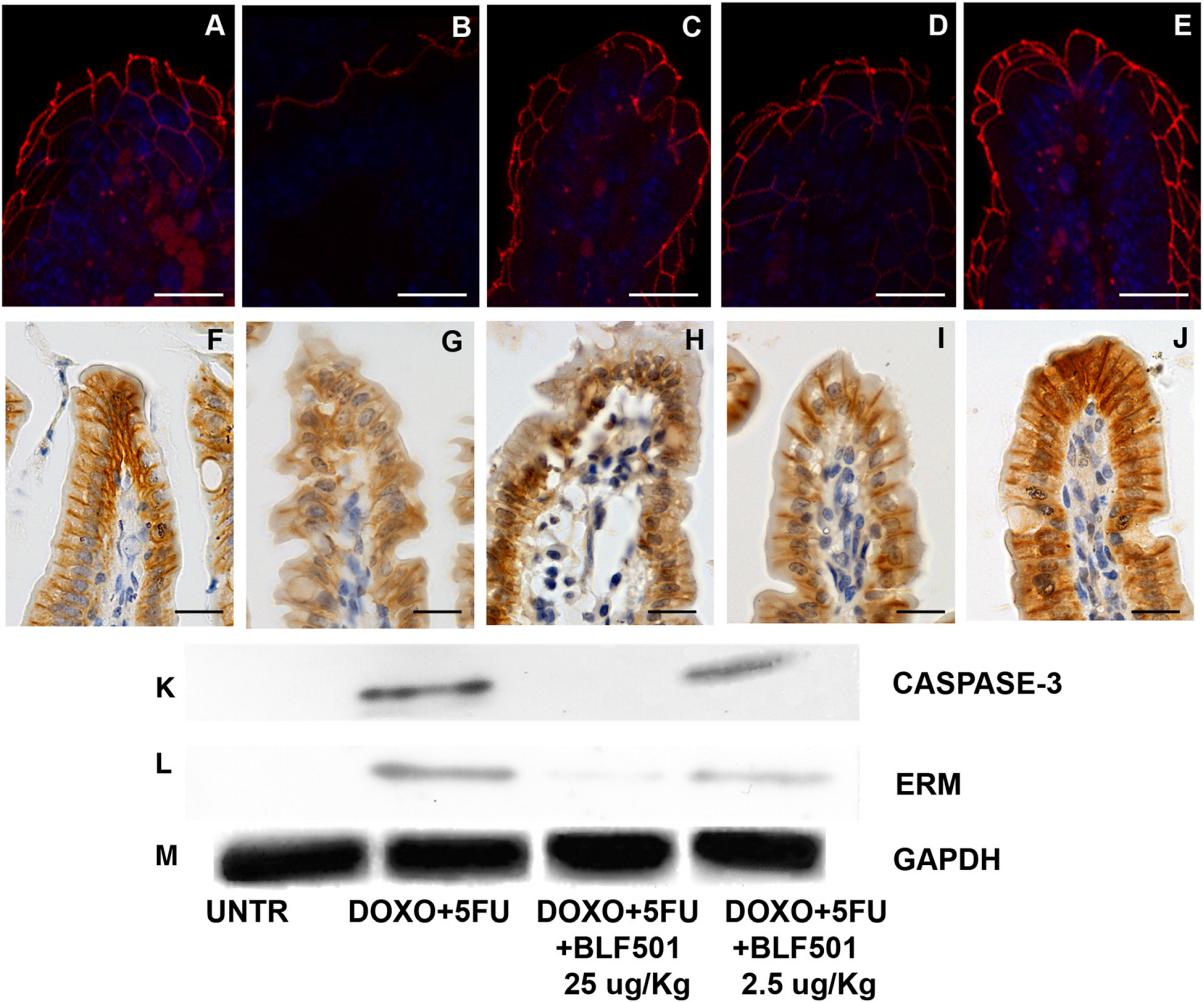
**Preservation of tight junction and adherence junction integrity by BLF501 in mice repeatedly injected with DXR and 5FU, stabilizing ZO-1 and beta-catenin expression and distribution.** ZO-1 immunofluorescence assay: **(A)** UNTR, untreated; **(B)** DXR + 5-FU; **(C)** BLF501 25 μg/kg; **(D)** DXR + 5-FU + BLF501 2.5 μg/kg; **(E)** DXR + 5-FU + BLF501 25 μg/kg. Magnification 60X. Immunohistochemical analysis of beta-catenin: **(F)** UNTR, untreated; **(G)** DXR + 5-FU; **(H)** DXR + 5-FU + BLF501 2.5 μg/kg; **(I)** DXR + 5-FU + BLF501 25 μg/kg; **(J)** BLF501 25 μg/kg. Magnification 40X. Western blot assay for epithelial damage markers caspase 3 **(K)**, ERM complex **(L)**, and GAPDH as housekeeping protein **(M)**. Experiments were performed in triplicate.

To examine the junctional systems of the intestinal epithelia of mice treated with the chemotherapeutics alone or together with BLF501, we focused on the expression of ZO-1, which mirrors the integrity of tight junctions [31,32], and beta-catenin, a component of adherens junctions [33]. Immunofluorescence and immunohistochemical staining of junctional systems revealed decreased expression of ZO-1 and an altered distribution of beta-catenin, respectively, in intestinal samples from DXR/5-FU-treated mice, whereas samples from mice that also received BLF501 at 25 μg/kg showed the typical honeycomb distribution of ZO-1 and expression/distribution of beta-catenin similar to that in control mice; the 2.5 μg/kg BLF501 dose was less effective. BLF501 (25 μg/kg) alone did not alter the expression or distribution of either ZO-1 or beta-catenin (Figure 5A-J).

We then evaluated extracts of small intestine samples for the expression of ERM proteins, which play a crucial role in organizing membrane domains through their ability to interact with transmembrane proteins and cytoskeleton [34], and caspase-3, an apoptosis marker whose expression is increased by chemotherapeutic treatment [35]. Administration of DXR and 5-FU induced overexpression of ERM proteins and caspase-3, whereas co-administration of BLF501 (25 μg/kg) reduced caspase-3 expression and restored normal levels of expression of ERM proteins. Protein expression was normalized to that of GAPDH (Figure 5K-M).

### Oral administration of BLF501 does not interfere with the antitumor activity of DXR

**Figure 6:**
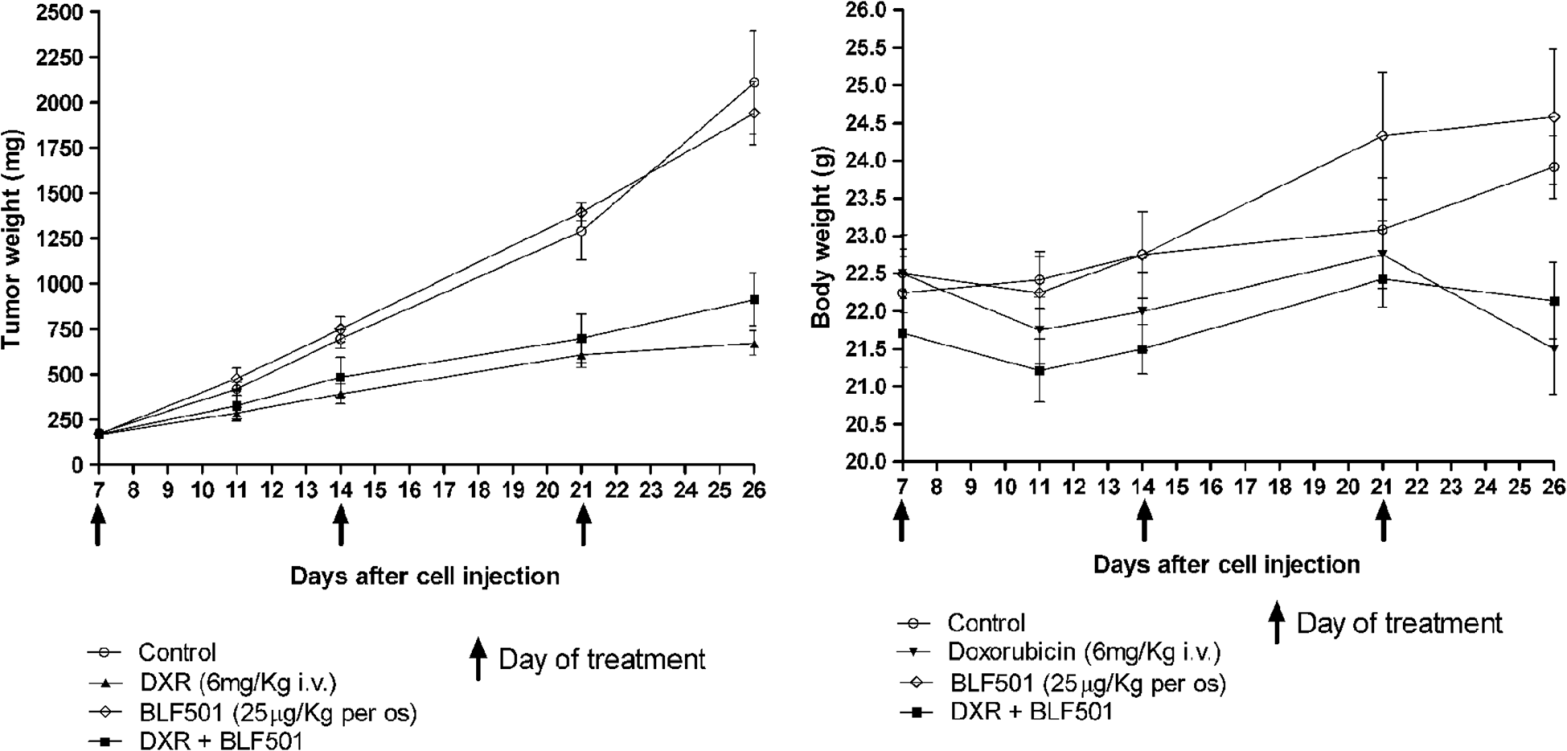
**Tumor growth rate and weight in mice treated with chemotherapy alone or in conjunction with BLF501.** Oral administration of BLF501 did not interfere with the antitumor activity of DXR. DXR-treated mice showed a reduction of body weight at the end of experiment, while a slight weight increase was observed in mice treated with DXR and BLF501. Error bars means SD Experiments were performed in triplicate.

Overexpression of SGLT-1 is a survival strategy utilized by several tumor types, including EGFR-positive tumors [36]. Analysis of athymic (nude) mice injected subcutaneously with A431 cells, which strongly express both EGFR and SGLT-1 [37], showed that the tumor growth rate in mice co-treated with both DXR and BLF501 was similar to that in mice treated with DXR alone (p = 0.1836) (Figure 6), indicating that oral administration of BLF501 does not interfere with DXR antitumor activity. Interestingly, while DXR-treated mice showed an average reduction of body weight at the end of experiment, a slight increase in weight was observed in the group of mice treated with DXR and BLF501. No differences in tumor growth rate were observed between untreated mice and mice treated with BLF501 alone.

## Discussion

The present data indicate that engagement of SGLT-1 by the synthetic D-glucose analog BLF501 promotes the protection of intestinal epithelial structures from injury induced by DXR and 5-FU. This protective effect is independent of glucose metabolism, since BLF501 is a C-glycoside and, as such, does not enter the metabolic pathways of D-glucose [21]. The protective effect of the orally-administered SGLT-1 agonist 3-O-methyl-glucose (3-OMG), a non-metabolizable analog of D-glucose that does not enter glucose metabolic pathways, and of D-glucose has been previously reported in a model of LPS-induced injury to the intestinal mucosa [19]. It is noteworthy that the dose of BLF501 necessary to protect from DXR-induced injury was much lower than doses of D-glucose or 3-OMG shown to protect from LPS-induced injury (25 μg/kg *vs* 2.5 g/kg). The ability of BLF501 to protect at low doses may reflect its high affinity for SGLT-1, a possibility supported by our previous *in vitro* finding that both D-glucose and BLF501 block LPS-induced release of IL-8, yet BLF501 is active at doses ~106-fold lower than that of D-glucose [21].

Regarding the mechanism underlying the therapeutic effect of BLF501, oral administration indirectly protects the intestinal epithelium from chemotherapy-induced injury which was detectable at 48 h and was almost complete at 72 h after a single administration of DXR. Cellular proliferation assays performed on SGLT-1-/- mice demonstrate the pivotal role of SGLT-1 engagement by BLF501 to exert ligand biological activity; in fact, in the absence of SGLT-1, no maintenance of proliferative processes was detected after BLF501 administration. Differences between the mean values of cell proliferation detected in wild-type and SGLT-1-/- mice probably reflect existing physiological differences between the BALB/C and C57 strains or an altered metabolism in SGLT-1-/- *vs* wild-type mice.

Among the different modifications observed upon co-administration of BLF501 with chemotherapeutics, we found reduced expression of caspase-3 in the intestines of co-treated mice compared to those treated with chemotherapeutics alone. This is likely due to a decrease in apoptotic events, consistent with recent reports that glucose administration reduces LPS-induced apoptotic events in enterocytes both *in vitro* and *in vivo*[16,38]. We also observed maintenance of the integrity of tight junctions in intestinal epithelia upon administration of BLF501 in mice treated with DXR and/or 5-FU, consistent with previous studies demonstrating that in the presence of heat- or chemical-induced sub-lethal stress conditions, activation of SGLT-1 preserves the integrity of tight junctions [16,18]. In particular, small intestine samples from mice treated with DXR and 5-FU showed altered expression and distribution of the junctional proteins ZO-1 and beta-catenin, whereas in mice co-treated with BLF501, ZO-1 and beta-catenin were normally expressed and distributed. Moreover, the ERM complex, which has a physiological role in the remodeling of junctional mechanisms [17,39-43], overexpressed upon treatment of DXR and 5-FU treatment causing the opening of junctional systems; BLF501 co-treatment maintained ERM expression into normal levels in mice co-treated with BLF501.

In our mucositis models, BLF501 was found to preserve proliferation and integrity of junctional systems, as well as to reduce the expression of a marker of apoptosis. These beneficial effects on the overall integrity of the epithelium were confirmed histologically. These findings strongly favor the use of BLF501 as a therapeutic tool for the maintenance of the integrity of the intestinal epithelium in the setting of chemotherapy-induced injury. In fact, chemotherapeutics induce apoptotic cell death and inhibit proliferation in rapidly dividing epithelia such as the intestinal epithelium [44]. As a consequence, mucosal atrophy and a reduction of the absorption capacity of the intestine ensue, leading to further deterioration of the general condition of patients who are already heavily compromised.

**Figure 7:**
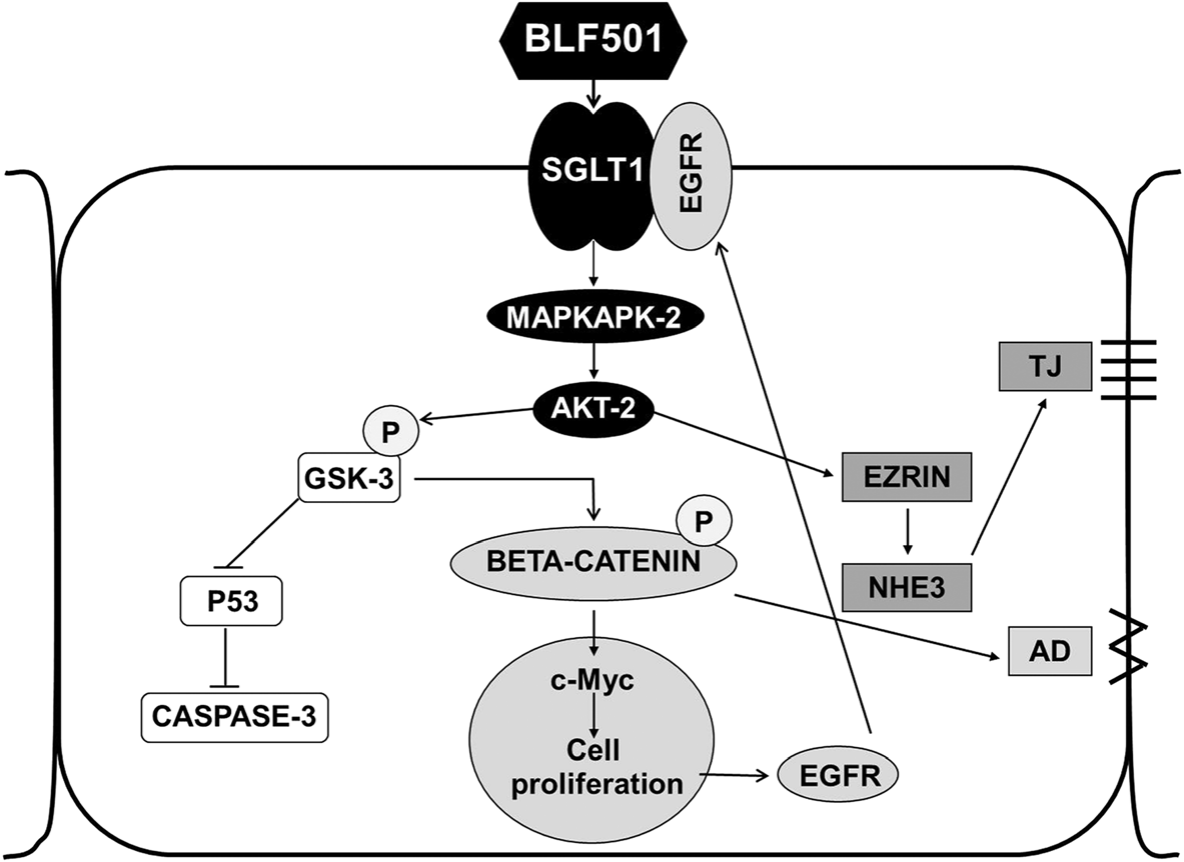
**Hypothetical cellular pathways activated by BLF501-mediated SGLT-1 engagement.** MAPKAPK-2/AKT-2 activation induces GSK-3 phosphorylation and consequent inhibition of p53–mediated caspase-3 cleavage. P-GSK-3 also preserves the cytoplasmic pool of beta-catenin involved in cellular proliferation mechanisms. Akt-2 is a key effector involved in tight junction management *via* ERM complex modulation.

The mechanism of interaction between SGLT-1 and its ligand BLF501 remains unknown. However based on several published findings [42,45,46] and as schematized in Figure 7, we hypothesize that BLF501-induced SGLT-1 engagement initiates downstream cellular signaling involving MAPKPK-2 and AKT-2 with consequent GSK-3 phosphorylation. P-GSK-3 acts to inhibit p53-induced casapse-3 cleavage and to preserve the phosphorylated beta-catenin cytoplasmic pool, with positive effects on cellular proliferation mechanisms. At the same time, AKT-2 activation modulates tight junction expression *via* the ERM complex. Clarification of the cellular pathways involved in the protective activity of BLF501 awaits further in-depth analyses.

In conclusion, our results show that oral administration of the non-metabolizable glucose analog BLF501 protects the intestinal mucosa from injuries induced by chemotherapeutic drugs. This suggests the prophylactic and/or therapeutic promise of BLF501 for the prevention or reduction of the severity of chemotherapy-induced mucositis. Moreover, orally administered BLF501 does not appear to interfere with the antitumor activity of the chemotherapeutics, with no difference in tumor growth between mice treated with DXR or DXR plus BLF501.

## Materials and methods

### Cells and culture conditions

A431 cells were purchased from ATCC (Rockville, MD) and authenticated using a panel of microsatellite markers (Istituto Nazionale Tumori, Milano, Italy). Cells were cultured in RPMI 1640 medium (Euroclone, Pero, Italy) supplemented with 10% FBS (Euroclone), 1% glutamine (Euroclone) and 1% penicillin/streptomycin solutions (Euroclone). A431 cells in log phase were digested and cell suspensions were inoculated subcutaneously under sterile conditions into mice.

### C-glycoside BLF501

The synthesis and a preliminary profiling of BLF501 have been described [21]. BLF501 resembles the structure (isosteric) of the natural O-glycoside, but cannot be metabolized (C-glycosides are unable to undergo glycolysis). BLF501 is water-soluble (5 mM) and stable for several days at 25°C at pH 1-12 (stored in the dark).

### Mice and *in vivo* treatments

Eight-week-old female BALB/c and nude SKH-1 mice were purchased from Charles River Italy (Calco, Italy). SGLT-1^-/-^ mice on a C57BL/6 background have recently been described [17]. Mice were housed in specific aseptic conditions at constant temperature and humidity, with food and water given *ad libitum*. Mice were fed a specific normocaloric chow which does not contain glucose and galactose (Altromin C1000, Rieper, BZ, Italy). Experimental protocols were approved by the Ethics Committee for Animal Experimentation of the Istituto Nazionale Tumori (Milano, Italy), and conducted according to the guidelines of the United Kingdom Coordinating Committee on Cancer Research for animal welfare in experimental neoplasia (1998).

The effect of BLF501 on intestinal injury induced by a single injection of DXR (Pfizer; NY, USA) was evaluated in four groups of BALB/C mice: controls (n = 7); DXR (n = 14); DXR plus BLF501 (n = 14); and BLF501 (n = 14). DXR (20 mg/kg in saline, total volume 300 μl) was administered by i.p. injection. BLF501 (25 μg/kg in saline, total volume 100 μl) was administered at the same time by gavage using a gastric tube. Control mice were treated i.p. with saline. One hour before sacrifice, bromodeoxyuridine (BrdU, Novocastra, Newcastle, UK) was injected i.p to determine cell proliferation. Control mice were sacrificed after 72 h. Mice from the other groups were sacrificed at 48 (n = 7) and at 72 h (n = 7) after treatment (Table 1) [47]. SGLT-1^-/-^ mice were randomly divided into three groups: UNTR; DXR; and DXR + BLF501 25 μg/kg. (n = 7/group) (Table 1) and sacrificed after 72 h.

The effect of BLF501 on intestinal injury induced by repeated injections of DXR and 5-FU (TEVA; Petah Tikva, Israel) was evaluated in six groups (n = 7/group): controls; DXR/5 + FU; DXR/5 + FU + BLF501 (0.25 μg/kg); DXR + 5-FU + BLF501 (2.5 μg/kg); DXR + 5-FU + BLF501 (25 μg/kg); BLF501 (25 μg/kg). DXR (7 mg/kg) and 5-FU (100 ng/kg), dissolved in saline solution in a final volume of 300 μl, were administered i.p. once per week for 3 weeks. BLF501 (in saline, 100 μl final volume) was administered at the same time by gavage using a gastric tube. Mice were sacrificed on day 19 after starting treatment (Table 1).

The effect of BLF501 on the antitumor activity of DXR was tested in four groups (n = 8/group) of nude SKH-1 mice bearing SGLT-1-positive A431 mammary tumors that had reached a mean weight of 240 mg: controls; DXR (6 mg/kg in 200 μl saline) + BLF501 (25 μg/kg in 100 μl saline); DXR alone; BLF501 alone. DXR was administered intravenously (i.v.) once per week for 3 weeks. BLF501 was administered at the same time by gavage using a gastric tube. Mice were sacrificed on day 26 of treatment (Table 1). Statistical significance was assessed using χ^2^ analysis.

### Processing of samples and histological evaluation

Mice were sacrificed and jejunum samples were collected and fixed in 10% formalin with 2% sucrose in phosphate buffer for 4 h at 4°C and processed for paraffin embedding. Other jejunum samples were collected and preserved in liquid nitrogen for mRNA and protein extraction.

For histological examination, slides were stained with hematoxylin and eosin. Histological images were captured and digitized. Villus height was measured using Image Pro Plus 4 image analysis software (Media Cybernetics, Baltimore, MD). The degree of intestinal tissue injury was evaluated on a grading scale of : +++ = severe; ++ = mild; + = light; 0 = absent.

### ZO-1 immunofluorescence

Briefly, sample sections on slides were deparaffinized and hydrated for 1 h through a descending scale of alcohols. After a quick rinse with 0.1 M Tris-HCl, pH 7.4, sections were incubated with proteinase K (20 g/ml) Tris-EDTA buffer, pH 8, for 15 min at 37°C, washed with Tris-HCl and permeabilized with 1% Triton X-100 in Tris-HCl for 5 min. Sections were treated with blocking solution for 1 h at room temperature and incubated overnight at 4°C with 20 μg/ml primary anti-mouse ZO-1 rabbit antibody (α-ZO-1, Invitrogen, Camarillo, CA). After washing, samples were incubated with secondary goat anti-rabbit antibody conjugated to tetramethyl-rhodamine isothiocyanate (TRITC; DyLight Jackson, West Baltimore Pike West Grove, PA, US) diluted 1:1000 in Tris-HCl for 45 min at room temperature. Sections were incubated with DAPI (1:10000 in Tris-HCl) for 5 min at room temperature and washed 3 times with Tris-HCl and 0.01% Triton X-100. Slides were mounted with Mowiol.

### Beta-catenin immunohistochemistry

Sample sections on slides were deparaffinized and hydrated for 1 h through a descending scale of alcohols. Antigen retrieval was performed using two antigen unmasking steps of 5 min in a microwave oven with citrate buffer, pH 6 (0.005 M). Sections were cooled and then washed with 0.1 M Tris-HCl, pH 7.4, + 0.025% Triton X-100. Samples were treated with a peroxidase inhibition solution of 3% H_2_O_2_ in 0.1 M Tris-HCl, pH 7.4, for 20 min and nonspecific sites were blocked with HHG solution (1 mM Hepes, 2% goat serum, 1X HBSS, 0.5% Triton X-100) in Tris-HCl for 1 h at room temperature. Sections were then incubated for 2 h at room temperature with a primary anti-beta-catenin rabbit antibody diluted 1:500 (Abcam, Cambridge, UK), washed, and incubated for 45 min at room temperature with a biotinylated secondary goat anti-rabbit antibody diluted 1:1000. Finally, sections were incubated with ABC-kit and DAB (Vector, Burlingame, VT, US), counterstained with hematoxylin, dehydrated through an ascending scale of alcohols and xylene, and mounted with coverslips using Entellan (Merck, Darmstadt, Germany).

### BrdU immunofluorescence

Sample sections on slides were deparaffinized and hydrated for 1 h through a descending scale of alcohols, rinsed in PBS and incubated at room temperature with 2 N HCl for 30 min and with Na_2_B_4_O_7_ for 10 min. Sections were incubated with PBS/3% BSA for 20 min at room temperature and with proteinase K (20 g/ml) Tris-EDTA buffer, pH 8, for 15 min at 37°C. After washing, slides were incubated for 1 h at room temperature with a primary anti-BrdU mouse antibody (Novocastra) diluted 1:200 in PBS/3% BSA, washed, and incubated with secondary goat anti-mouse DyLight 488 antibody (Jackson) diluted 1:500 in PBS/BSA 3%. Sections were washed, incubated for 5 min at room temperature with DAPI diluted 1:2500 in PBS, and mounted with Mowiol.

All samples were observed and photographed with a microscope Nikon Eclipse 80 with a digital camera Nikon DS-L1.

### Real-time polymerase-chain reaction (PCR)

RNA was extracted from frozen jejunum samples using TRI-reagent (Sigma-Aldrich, St. Louis, MO, US) and transcribed with a reverse transcription kit (Applied Biosystems, Foster City, CA, US). Real-time PCR experiments were performed according to the manufacturer’s instructions using a 7900HT Fast Real-Time PCR System (Applied Biosystems). Primers for SI, TFF3, DLL1, beta-actin and the housekeeping gene 18S were purchased from Applied Biosystems.

### Western blot analysis

Expression of caspase-3 and ERM complex was assessed on total phosphorylated protein extracted from small intestine samples of mice using our TNTG buffer (40 mM Tris, pH 7.5, 100 mM NaCl, 10% glycerol, 1% Triton X-100, proteases inhibitors). Protein extracts were quantified using the BCA method (BCA Protein Assay Kit, Pierce, Rockford, IL, US). Proteins (30 μg) were fractionated on a polyacrylamide gel (BIO-RAD Labs, Hercules, CA) and electroblotted onto nitrocellulose filters (American Biosciences, Buckinghamshire, UK). Filters were incubated for 1 h in TBS containing 5% milk powder to block nonspecific binding sites, followed by anti-caspase-3 antibody (1 μg/ml; Abcam), anti-GAPDH antibody (1:1000; Abcam) and anti-ERM antibody (1:1000; Cell Signaling Technologies, Danvers, MA, US), and finally with appropriate anti-goat and anti-rabbit secondary anti-peroxide antibody (Vector Laboratories). Bands were visualized using ECL™ Western Blotting Detection Reagents and plates by autoradiography (American Biosciences).

### Statistical analyses

Student’s t-test (paired two-tailed), χ^2^ analysis and GraphPad Prism software (GraphPad Prism Software Inc., San Diego, CA) were used for comparisons between groups. Differences were considered significant at p < 0.05.

## Competing interests

The authors declare that they have no competing interests.

## Authors’ contributions

DC and CR made initial observations, designed the main experiments, analyzed the data and wrote the manuscript; BLF and GD designed and performed the synthesis of BLF501 molecule; DC, CS and MS performed all of the studies involving animal models, including histological and immunohistochemical analyses, biochemical assays and molecular biology experiments, and contributed to manuscript preparation; DO performed all anatomopathological analysis; FM, AB, FN, and ET supervised the project and data interpretation and contributed to manuscript preparation; HK make the SGLT-1 KO mice available and contributed to data interpretation; CR designed the experimental approach, coordinated the project and wrote the manuscript. All authors have read and approved the final manuscript.
